# Microwave-Assisted Hydrothermal Synthesis of Cellulose/Hydroxyapatite Nanocomposites

**DOI:** 10.3390/polym8090316

**Published:** 2016-09-20

**Authors:** Lian-Hua Fu, Yan-Jun Liu, Ming-Guo Ma, Xue-Ming Zhang, Zhi-Min Xue, Jie-Fang Zhu

**Affiliations:** 1 Beijing Key Laboratory of Lignocellulosic Chemistry, College of Materials Science and Technology, Beijing Forestry University, Beijing 100083, China; fly2016@bjfu.edu.cn (L.-H.F.); yj_liu@bjfu.edu.cn (Y.-J.L.); xm_zhang@bifu.edu.cn (X.-M.Z.); zmxue@bjfu.edu.cn (Z.-M.X.); 2 Department of Chemistry-Ångström Laboratory, Uppsala University, Uppsala 75121, Sweden

**Keywords:** cellulose, hydroxyapatite, nanocomposites, microwave-assisted hydrothermal

## Abstract

In this paper, we report a facile, rapid, and green strategy for the synthesis of cellulose/hydroxyapatite (HA) nanocomposites using an inorganic phosphorus source (sodium dihydrogen phosphate dihydrate (NaH_2_PO_4_·2H_2_O)), or organic phosphorus sources (adenosine 5′-triphosphate disodium salt (ATP), creatine phosphate disodium salt tetrahydrate (CP), or D-fructose 1,6-bisphosphate trisodium salt octahydrate (FBP)) through the microwave-assisted hydrothermal method. The effects of the phosphorus sources, heating time, and heating temperature on the phase, size, and morphology of the products were systematically investigated. The experimental results revealed that the phosphate sources played a critical role on the phase, size, and morphology of the minerals in the nanocomposites. For example, the pure HA was obtained by using NaH_2_PO_4_·2H_2_O as phosphorus source, while all the ATP, CP, and FBP led to the byproduct, calcite. The HA nanostructures with various morphologies (including nanorods, pseudo-cubic, pseudo-spherical, and nano-spherical particles) were obtained by varying the phosphorus sources or adjusting the reaction parameters. In addition, this strategy is surfactant-free, avoiding the post-treatment procedure and cost for the surfactant removal from the product. We believe that this work can be a guidance for the green synthesis of cellulose/HA nanocomposites in the future.

## 1. Introduction

Calcium phosphate materials have received great interest, since most of them are widely investigated for applications in biomedical fields, due to their excellent biocompatibility, osteoconductivity, bioactivity, and similarity to the inorganic component of natural bone [1,2,3]. Among various calcium phosphate materials, hydroxyapatite (Ca_10_(PO_4_)_6_(OH)_2_, HA) is the main inorganic component of vertebrate bones and teeth as well as the most stable calcium phosphate crystalline phase under physiological conditions [4,5,6]. In biological systems, the nucleation and growth of biological apatite often occur in an environment full of various ions, resulting in the inner crystalline disorder and presence of other ions in the crystal lattice of biological apatite. Moreover, biological apatite often exists as carbonated apatite with nonstoichiometry composition, low crystallinity, and small dimensions, which also increase its solubility when compared with chemically pure HA [5,6,7]. For decades, synthetic HA has been recognized as an excellent candidate for biomedical applications, such as bone tissue engineering [8,9], imaging and diagnosis materials [10,11], and protein/drug/gene delivery [12,13,14], owing to its excellent biocompatibility, affinity to biopolymers, nontoxicity, high osteogenic potential, and high chemical and thermal stability [15,16,17].

Cellulose, as the most abundant natural renewable polysaccharide on the earth [18], has been receiving considerable attention for use in the preparation of organic/inorganic composites owing to its unique properties, including nontoxicity, biocompatibility, chemical stability, mechanical strength, and biodegradation [19]. The cellulose/HA nanocomposites, resulting from the incorporation of HA nanoparticles into cellulose substrate, are considered to be interesting functional materials with many potential applications in various fields, such as water treatment [20,21,22], bone tissue engineering [23,24,25,26], and protein adsorption [27]. The HA nanostructures with high specific surface area and unsaturated atoms could interact with cellulose substrate, leading to the enhanced properties of their composites [28]. For example, the cellulose/HA nanocomposites prepared via in situ hybridization were found to efficiently remove fluoride from drinking water, due to a higher adsorption capacity of fluoride, compared with the individual HA nanoparticles [20]. The HA microfibrillated cellulose composites were used as an adsorbent for removal of Cr(VI) from aqueous solution [21]. The bacterial cellulose (BC)/HA nanocomposites were synthesized using a biomimetic approach, and the presence of HA crystals on BC surfaces increased cell attachment [24]. Favi et al. prepared the BC scaffolds first, which were then mineralized with nano-HA to mimic the inorganic component of native bone tissue. The as-obtained HA/BC composites with well-defined honeycomb pore arrays were proved promising for bone tissue engineering applications [25]. In addition, cellulose triacetate (CTA) nanofibers decorated by HA nanoparticles were used for bovine serum albumin (BSA) adsorption, and the maximum equilibrium adsorption capacity for BSA was 176.04 mg·g^−1^, much higher than CTA nanofibers (18.39 mg·g^−1^) [27].

Conventionally, the cellulose/HA nanocomposites are prepared by using inorganic phosphorus sources, such as phosphorus ions [20,21,24,25,26,28]. The preparation of cellulose/HA nanocomposites using organic phosphorus sources such as phosphorus-containing biomolecules has been rarely reported. Compared with the inorganic phosphorus source such as phosphate salts, using phosphorus-containing biomolecules as organic phosphorus sources to synthesize cellulose/HA nanocomposites has several advantages. For example, the phosphorus source existing in the form of phosphate groups in organic biomolecules can avoid the fast nucleation and disordered growth of the product. Phosphorus-containing organic biomolecules generally require certain conditions (such as heating in aqueous solution) to hydrolyze to phosphate ions, the rate of which can be controlled to determine the size, morphology, and structure of the product. Furthermore, organic biomolecules are essentially nontoxic and biocompatible.

Adenosine 5′-triphosphate disodium salt (ATP)—the energy carrier of cells in biological systems, containing three phosphate groups in one molecule—has been demonstrated as a promising organic phosphorus source for the preparation of phosphate biomaterials [29,30]. Creatine phosphate disodium salt tetrahydrate (CP) is formed after phosphorylation of creatine by creatine kinase in the liver [31], which can hydrolyze to form PO_4_^3−^ ions in aqueous solution under heating conditions [7,32]. In addition, the D-fructose 1,6-bisphosphate trisodium salt octahydrate (FBP) biomolecule can hydrolyze to produce PO_4_^3−^ ions and fructose molecules in a controlled manner under heating conditions [33,34]. In this study, the cellulose/HA nanocomposites were synthesized by using the phosphate salt (NaH_2_PO_4_) as an inorganic phosphorus source, or the biocompatible phosphorus-containing biomolecules (ATP, CP, or FBP) as organic phosphorus sources through a microwave-assisted hydrothermal method. The microwave-assisted heating method has many advantages over conventional heating methods, such as higher reaction rate, shorter reaction time (usually in min rather than h or days), reduced energy consumption, and environmental friendliness [35], which has been a fast-growing area in materials synthesis [36,37]. Here, the universality of microwave-assisted synthesis can also be proved by using different phosphorus sources. The effects of phosphorus source, heating time, and temperature on the phase, size, and morphology of the products were systematically investigated. The experimental results revealed that phosphate sources played a vital role on the formation of the cellulose/HA nanocomposites. It should be highlighted that the method reported here is facile, rapid, surfactant-free, and environmentally friendly.

## 2. Experimental Section

### 2.1. Materials

**Figure 1:**
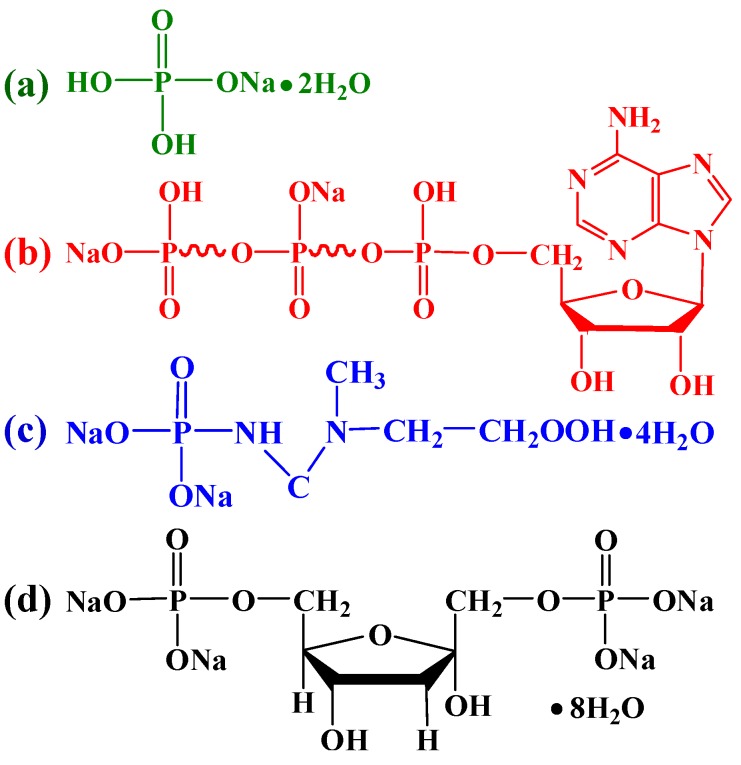
Chemical structures of the phosphate salt and biocompatible phosphorus-containing biomolecules used in this study: (**a**) sodium dihydrogen phosphate dihydrate (NaH_2_PO_4_·2H_2_O); (**b**) adenosine 5′-triphosphate disodium salt (ATP); (**c**) creatine phosphate disodium salt tetrahydrate (CP); (**d**) D-fructose 1,6-bisphosphate trisodium salt octahydrate (FBP).

Microcrystalline cellulose (molecular weight of 34,033–38,894, degree of polymerization (DP) = 210–240) was purchased from Sinopharm Chemical Reagent Co., Ltd. (Shanghai, China). Adenosine 5′-triphosphate disodium salt (ATP, *M*_w_ = 551.14), and creatine phosphate disodium salt tetrahydrate (CP, *M*_w_ = 327.13) were purchased from J&K China Chemical, Ltd. (Beijing, China). D-fructose 1,6-bisphosphate trisodium salt octahydrate (FBP, *M*_w_ = 406.06) was purchased from Aladdin Industrial Co. (Shanghai, China). Anhydrous calcium chloride (CaCl_2_, *M*_w_ = 110.98), sodium dihydrogen phosphate dihydrate (NaH_2_PO_4_·2H_2_O, *M*_w_ = 156.01), and sodium hydroxide (NaOH, *M*_w_ = 40.00) were purchased from Beijing Chemical Works (Beijing, China). Urea (CH_4_ON_2_, *M*_w_ = 66.06) was purchased from Xilong Chemical Co., Ltd. (Shantou, China). Figure 1 displays the chemical structures of the NaH_2_PO_4_, ATP, CP, and FBP. All chemicals in this work were of analytical grades and used as received without further purification. All solutions were prepared by ultrapure water (*R* > 18 MΩ·cm).

### 2.2. Preparation of the Cellulose/HA Nanocomposites

In a typical experiment, for the preparation of cellulose solution, 7.00 g NaOH and 12.00 g urea were added into 81 mL deionized water to form NaOH–urea aqueous solution. Then, 1.62 g microcrystalline cellulose was added into the above solution under magnetic stirring (600 rpm, 10 min). After that, the above solution was cooled to −12 °C for 12 h. For the synthesis of cellulose/HA nanocomposites, 10 mL CaCl_2_ (0.1 M) was highly dispersed in the obtained cellulose solution (10 mL). Then, 10 mL NaH_2_PO_4_·2H_2_O, ATP, CP, or FBP, at molar ratio Ca/P = 1.67, was added in the resulting colloidal solution. The solution was loaded into an autoclave (60 mL), sealed, and heated in a microwave oven (MDS-6G, Sineo, Shanghai, China) to the temperature of 150 or 180 °C for 10 or 60 min. After being cooled down naturally to room temperature, the product was separated by centrifugation (9000 rpm, 8 min), washed by deionized water until the supernatant was neutral, washed by ethanol for one time and dried in an oven at 60 °C for 24 h. After grinding the dried product in a ceramic mortar, the powder sample was obtained. Table 1 displays the detailed experiment parameters.

### 2.3. Characterization

X-ray powder diffraction (XRD) patterns of the as-prepared samples were recorded on Rigaku D/Max 2200-PC (Tokyo, Japan) in 2*θ* range from 10° to 70°, operating with Cu Kα (λ = 1.5405 Å) radiation at 40 kV and 20 mA with a step size of 0.02° at a scanning rate of 0.12° s^−1^. Fourier-transform infrared (FT-IR) spectra were obtained with Bruker VERTEX 70V (Karlsruhe, Germany). The spectra were recorded in the range of 4000–400 cm^−1^ at 4 cm^−1^ resolution and 64 scans per sample. Field emission scanning electron microscopy (FE-SEM) images were recorded with a Hitachi SU8010 (Tokyo, Japan) at 5 kV and 10 μA, and all samples were Au coated prior to examination by FE-SEM. The sizes of HA nanoparticles in the nanocomposites were obtained from the FE-SEM image using Image J software (NIH, Bethesda, MD, USA). The Ca/P molar ratio of the product was calculated from the Ca and P elemental contents in the sample, which were obtained using an inductively coupled plasma (ICP) optical emission spectrometer, Horiba JY2000-2 (Paris, France). Ten milligrams of the powder samples were dispersed in 5 mL HCl solution (0.1 M) by being shaken at a constant rate at 37 °C for 4 h to dissolve the samples completely. After centrifugation (9000 rpm for 8 min), the supernatant was measured by ICP to determine the contents of Ca and P. Thermogravimetric analysis (TGA) and derivative thermogravimetry (DTG) were taken with a heating rate of 10 °C·min^−1^ from room temperature to 800 °C in flowing air on a Shimadzu DTG-60 (Kyoto, Japan), and each sample was weighed between 3 and 5 mg for analysis.

## 3. Results and Discussion

**Figure 2:**
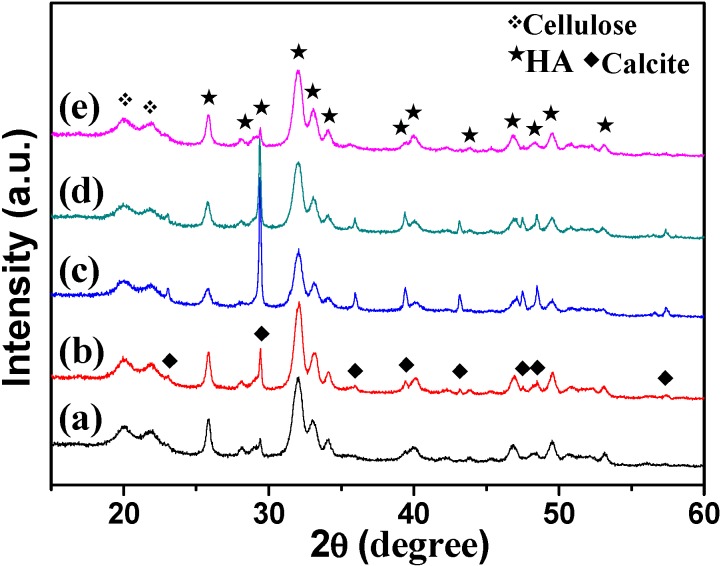
X-ray powder diffraction (XRD) patterns of the cellulose/HA nanocomposites prepared by microwave-assisted hydrothermal method at 150 °C for 10 min (**a**, **c**–**e**) and 60 min (**b**) using different phosphorus sources: (**a**, **b**) NaH_2_PO_4_·2H_2_O; (**c**) ATP; (**d**) CP; (**e**) FBP.

To determine the crystal phases of the as-prepared products, the XRD patterns of the samples were recorded. As shown in Figure 2, all of the samples show diffraction peaks at around 2*θ =* 20.03° and 21.89°, which were attributed to cellulose II (JCPDS 03-0226). The other peaks in the sample prepared using NaH_2_PO_4_·2H_2_O as phosphorus source through microwave-assisted hydrothermal method at 150 °C for 10 min were indexed to HA (JCPDS 09-0432) with hexagonal structure (Figure 2a). The Ca/P molar ratio was 1.82 (measured by ICP). Prolonging the heating time to 60 min (Figure 2b), the product contained HA and a small amount of calcite (JCPDS 47-1743), and the Ca/P molar ratio was increased to 2.00. The XRD patterns of the samples prepared using ATP (Figure 2c) and CP (Figure 2d) were similar to Figure 2b, while the sharp peaks at 2θ = 29.36°, 39.38°, 47.08°, and 48.46° suggest the higher crystallinity of calcite. The contents of calcite also increased, and the Ca/P molar ratios were 2.63 and 2.32, corresponding to the samples using ATP and CP, respectively. When using FBP as phosphorus source (Figure 2e), the diffraction peaks were similar to Figure 2a, and the Ca/P molar ratio was 1.87. It is clear that all the five samples possess high Ca/P molar ratios (1.82–2.63). This result can be explained by the following reasons: (1) The Ca and P contents analyzed by ICP were the total element contents in the minerals, including HA (Ca_10_(PO_4_)_6_(OH)_2_) and the byproduct calcite (CaCO_3_). The existence of calcite impurities contributes to the high Ca/P molar ratios. (2) The [PO_4_^3−^] in HA may be partly replaced by [CO_3_^2−^] from the urea (confirmed by the FT-IR spectra subsequently), and this replacement could also lead to high Ca/P molar ratios.

The XRD results of cellulose/HA nanocomposites were different from the previous reports, where the calcium phosphate materials (HA or CHA) without the impurities (calcite) were obtained y using inorganic phosphate salt (NaH_2_PO_4_) [19,37] or biocompatible phosphorus-containing biomolecules (ATP, CP, and FBP) [13,32,34]. The reasons for the differences can be explained as follows:

(i) In this study, the NaOH–urea aqueous solution not only served as the solvent for cellulose [38], but also provided the alkaline environment needed for the formation of HA. The urea (CO(NH_2_)_2_), as the diamide of carbonic acid (H_2_CO_3_), is thermally instable, which can be decomposed into melamine (C_3_H_6_N_6_), ammonia (NH_3_), and carbon dioxide (CO_2_) when being heated to 150 °C (Equation (1)). The gaseous products (NH_3_ and CO_2_) remained in the reaction system when the microwave-assisted hydrothermal heating was carried out in a closed autoclave. The CO_2_ would be converted into H_2_CO_3_ (Equation (2)), and the H_2_CO_3_ was ionized into CO_3_^2−^ (Equation (3)) under the strong alkaline condition.

(1) $$CO{\left(NH_{2}\right)}_{2}\overset{\;\Delta \;}{\to }C_{3}H_{6}N_{6}+6\;NH_{3}↑+3\;CO_{2}↑$$

(2) $$CO_{2}+H_{2}O\;\to \;H_{2}CO_{3}$$

(3) $$H_{2}CO_{3}\overset{\;OH^{-}\;}{\to }H_{2}O+{HCO_{3}}^{-}\overset{\;OH^{-}\;}{\to }H_{2}O+{CO_{3}}^{2-}$$

(ii) It is well known that the solubility product (*K*_sp_) of HA is far less than calcite under the same temperature. Therefore, the HA was formed preferentially in the reaction system (containing Ca^2+^, PO_4_^3−^, and CO_3_^2−^ under alkaline condition) and then the calcite. This can be responsible for the sample prepared using NaH_2_PO_4_·2H_2_O as the phosphorus source at 150 °C for 10 min (Figure 2a), where the mineral was HA without any impurity. However, when prolonging the heating time to 60 min, the formation of HA was accompanied by the decomposition of urea, and the pH value decreased gradually resulting in the increasing number of CO_3_^2^^−^ (see the Equations (1)–(3)). The existence of a large number of CO_3_^2−^ made it possible to capture the Ca^2+^, resulting in the appearance of the diffraction peaks of calcite (Figure 2b).

(iii) For the organic biomolecules ATP and CP, the phosphorus sources exist in the form of phosphate groups (Figure 1b,c), and the organic biomolecules generally require certain conditions to hydrolyze to phosphate ions, which can avoid the fast nucleation of the product [12,36]. For example, ATP can be hydrolyzed to phosphate group accompanied with the releases of a large amount of energy in the metabolism. In addition, in natural process, CP is generated from excess ATP during resting periods through the transport of high energy phosphate; also, the energy can be given off from donating the phosphate group [32]. However, the relatively slow hydrolysis rate of the biomolecules was just the reason that led to the formation of calcite (Figure 2c,d). As the product of the phosphorolysis of fructose, FBP biomolecules can be hydrolyzed to produce PO_4_^3^^−^ easier than ATP and CP. It suggests that calcite might also exist in the mineral, but it was too little to be detected by XRD.

**Figure 3:**
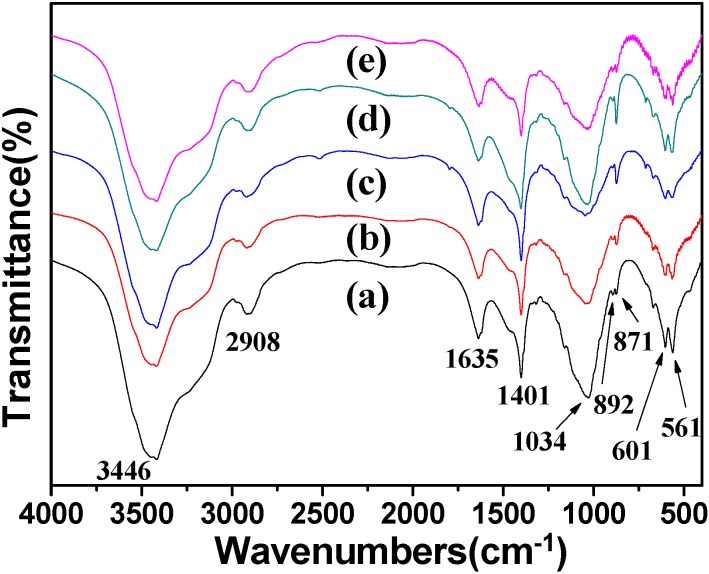
Fourier-transform infrared (FT-IR) spectra of the cellulose/HA nanocomposites prepared by microwave-assisted hydrothermal method at 150 °C for 10 min (**a**, **c**–**e**) and 60 min (**b**) using different phosphorus sources: (**a**, **b**) NaH_2_PO_4_·2H_2_O; (**c**) ATP; (**d**) CP; (**e**) FBP.

The results from XRD patterns are also in agreement with those from FT-IR spectra, as shown in Figure 3. The peaks at 3446 and 1635 cm^−1^ are attributed to the –OH and the bending mode of absorbed water in the cellulose/HA nanocomposites. The adsorptions at 2908 and 892 cm^−1^ belong to the stretching vibration of C–H groups in pyranoid ring and the *β*-glycosidic linkages in cellulose, respectively. The broad peak at 1034 cm^−1^ is attributed to the C–O–C pyranose ring skeletal vibration of cellulose or the *v*_3_ vibration of O–P in HA, and the peaks located at 601 and 561 cm^−1^ can be ascribed to the *v*_4_ bending mode of O–P–O. Sharp peaks appeared at 1401 (*v*_3-4_ CO_3_^2−^) and 871 cm^−1^ (*v*_2_ CO_3_^2−^) in all of the samples, suggesting the existence of calcite or the [PO_4_^3−^] being partly replaced by [CO_3_^2−^] from the urea [19,39]. The appearance of these two peaks of CO_3_^2−^ just confirmed the speculation about the high Ca/P molar ratios before. Based on the results of XRD and FT-IR analysis, one can draw the following conclusions: (1) The phosphorus sources affected the crystal phases of the minerals in the cellulose/HA nanocomposites; (2) The longer heating time could lead to byproduct (calcite). In another word, the shorter heating time favored the formation of HA in the cellulose/HA nanocomposites.

**Figure 4:**
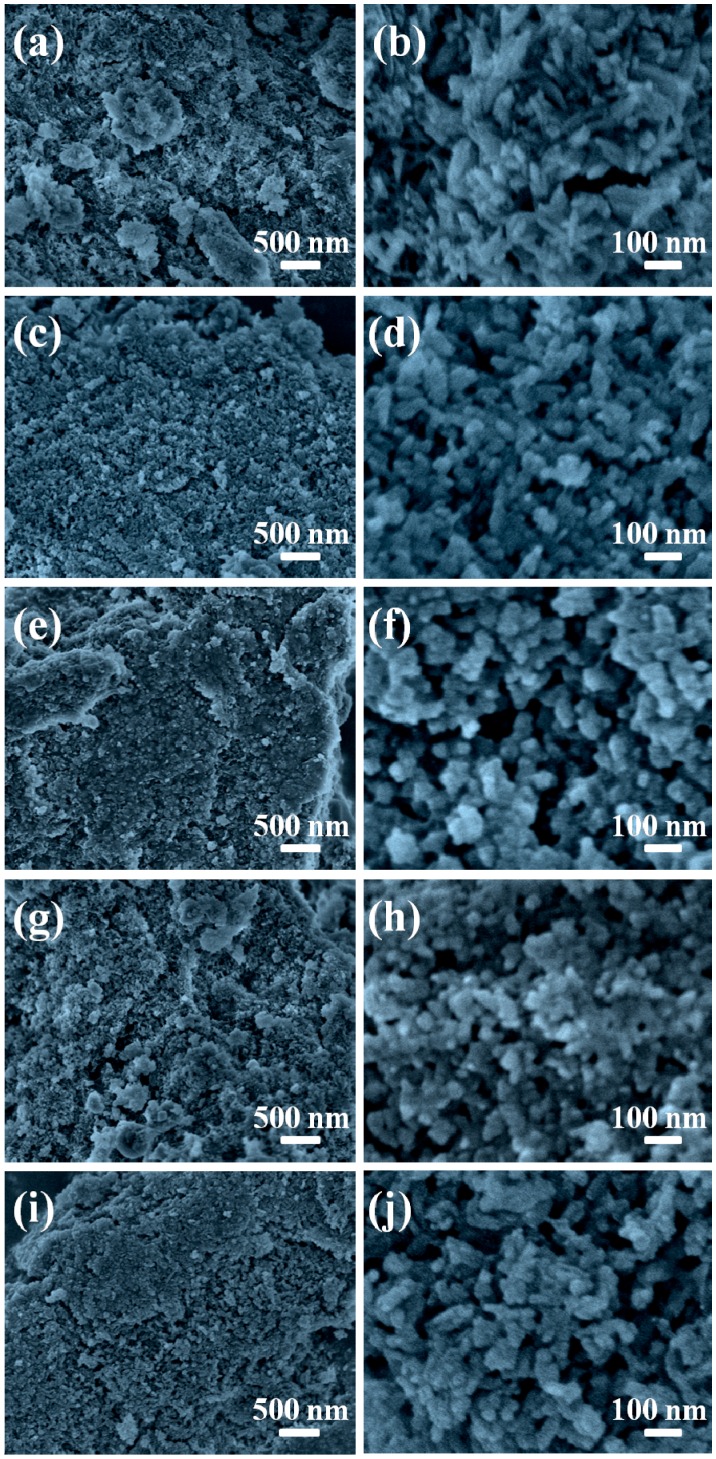
FE-SEM images of the cellulose/HA nanocomposites prepared by microwave-assisted hydrothermal method at 150 °C for 10 min using different phosphorus sources: (**a**, **b**) NaH_2_PO_4_·2H_2_O; (**e**, **f**) ATP; (**g**, **h**) CP; (**i**, **j**) FBP and using (**c**, **d**) NaH_2_PO_4_·2H_2_O at 150 °C for 60 min. The magnifications for the low-magnification images on the left column are 20,000 and 100,000 times for the high-magnification images on the right column.

The morphologies of the cellulose/HA nanocomposites were also investigated with FE-SEM, as shown in Figure 4. The cellulose substrates were covered by a large number of HA nanostructures in all five samples. The HA nanostructures in the cellulose/HA nanocomposites prepared using NaH_2_PO_4_·2H_2_O as phosphorus source at 150 °C for 10 min appeared as nanorods with 97.1 ± 9.4 nm in length and 21.6 ± 2.8 nm in diameter (Figure 4a,b). When increasing the heating time to 60 min (Figure 4c,d), the nanorods were shorter in length (61.2 ± 4.2 nm) and thicker in diameter (33.1 ± 4.1 nm). When using ATP as the organic phosphorus source (Figure 4e,f), the HA with pseudo-cubic structure (the size of the HA crystals was 48.1 ± 4.3 nm) was obtained. Similarly, the pseudo-spherical structure with smaller size of 28.8 ± 3.1 nm was obtained using CP as the organic phosphorus source (Figure 4g,h). When using FBP (Figure 4i,j), the HA nanostructures also appeared as nanorods (69.4 ± 9.3 nm in length and 26.9 ± 1.7 nm in diameter), while their surface became coarse. These results indicated that the phosphorus sources had effects on the size and morphology of HA in the nanocomposites, and the HA nanostructures with smaller size could be obtained by prolonging the reaction time.

**Figure 5:**
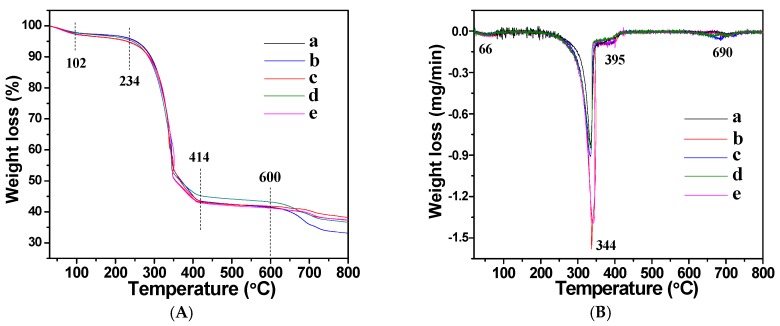
(**A**) Thermogravimetric analysis (TGA) and (**B**) derivative thermogravimetry (DTG) curves of the cellulose/HA nanocomposites prepared by microwave-assisted hydrothermal method at 150 °C for 10 min (**a**, **c**–**e**) and 60 min (**b**) using different phosphorus sources: (**a**, **b**) NaH_2_PO_4_·2H_2_O; (**c**) ATP; (**d**) CP; (**e**) FBP.

The thermal stability of the as-prepared cellulose/HA nanocomposites in air was investigated by thermogravimetric analysis (TGA) and derivative thermogravimetry (DTG), as shown in Figure 5. The TGA curves (Figure 5A) show slight weight losses around 102 °C, due to the loss of absorbed water in the nanocomposites, and the weight declined dramatically between 234 and 414 °C, ascribed to the degradation of the cellulose substrates and biocompatible phosphorus-containing biomolecules [13,34,40]. In addition, the weight loss from 600 to 800 °C was caused by the decomposition of calcite, since the complete decomposition of calcite occurs below 800 °C. The DTG curves (Figure 5B) show four peaks located at around 66, 344, 395, and 690 °C, which fit well with those of weight loss in the TGA curves (Figure 5A). The weight losses of the cellulose/HA nanocomposites in the temperature range investigated were about 61.8% (a), 62.8% (b), 66.9% (c), 63.4% (d), and 63.6% (e), respectively. These results indicated that the reaction time did not have obvious influence on the thermal stability of the nanocomposites, and the samples prepared using inorganic or organic phosphorus source had similar thermal stability, except ATP. Combining the XRD patterns (Figure 2) and the Ca/P molar ratio from ICP analysis (Table 1), the cellulose/HA nanocomposites prepared using ATP as phosphorus source may possess the maximum content of calcite with high crystallinity, leading to the highest weight loss among all the five samples.

**Figure 6:**
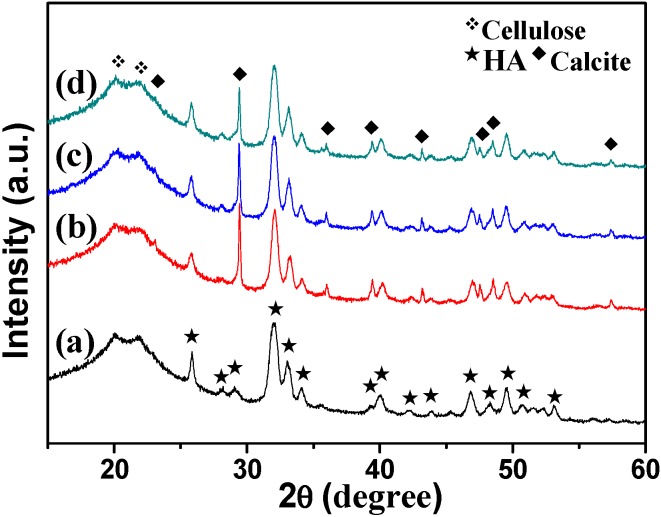
XRD patterns of the cellulose/HA nanocomposites prepared by microwave-assisted hydrothermal method at 180 °C for 10 min using different phosphorus sources: (**a**) NaH_2_PO_4_·2H_2_O; (**b**) ATP; (**c**) CP; (**d**) FBP.

Moreover, we investigated the effect of heating temperature on the formation of the cellulose/HA nanocomposites. As shown in Figure 6, the diffraction peaks of cellulose were located at around 2θ *=* 20.08° and 21.78° in all of the samples, and there was no obvious difference on the crystal phase of the cellulose/HA nanocomposites prepared using NaH_2_PO_4_·2H_2_O as phosphorus source at 180 °C for 10 min (Figure 6a), compared with that prepared at 150 °C for 10 min (Figure 2a). The cellulose/HA nanocomposites also consisted of majority phases of cellulose (JCPDS 03-0226) and HA (JCPDS 09-0432), and minority phase of calcite (JCPDS 47-1743) using ATP (Figure 6b) or CP (Figure 6c) as phosphorus sources, while the Ca/P molar ratios decreased from 2.63 to 2.33, and 2.32 to 2.17 (Table 1), respectively. These results indicate that the higher temperature accelerated the hydrolysis of the biomolecules, leading to more PO_4_^3^^−^ in the reaction system. On the other hand, the HA nuclei in the composites can be formed more rapidly at higher temperatures [39]. When using FBP (Figure 6d), the calcite (JCPDS 47-1743) was observed and the Ca/P molar ratio increased from 1.87 to 2.07, compared with the cellulose/HA nanocomposites prepared at 150 °C for 10 min. This result may result from the decomposition of FBP. Under microwave-assisted hydrothermal reaction condition, the FBP molecules could hydrolyze to form PO_4_^3−^ and D-fructose 1,6-bisphosphate (FDP), as shown in Equation (4). The FDP could decompose into CO_2_ and H_2_O in the presence of oxygen (O_2_) under higher temperature (180 °C), for it was thermally unstable, as shown in Equation (5). Then CO_2_ would be converted into CO_3_^2−^ following Equations (2) and (3), and the existence of a large number of CO_3_^2−^ made it possible to capture Ca^2+^. Hence, the diffraction peaks of calcite appeared (Figure 6d). However, this decomposition would be terminated for the limited O_2_ in the reaction system, owing to the microwave-assisted hydrothermal heating in a closed autoclave.

(4) $$FBP\to FDP+2\;{PO_{4}}^{3-}$$

(5) $$FDP+O_{2}\to CO_{2}↑+H_{2}O$$

**Figure 7:**
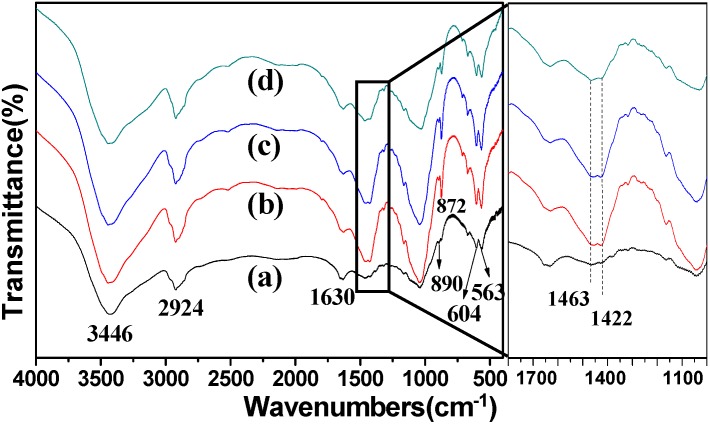
FT-IR spectra of the cellulose/HA nanocomposites prepared by microwave-assisted hydrothermal method at 180 °C for 10 min using different phosphorus sources: (**a**) NaH_2_PO_4_·2H_2_O; (**b**) ATP; (**c**) CP; (**d**) FBP.

The FT-IR spectra of the cellulose/HA nanocomposites prepared at 180 °C for 10 min using NaH_2_PO_4_·2H_2_O, ATP, CP, and FBP as phosphorus sources were exhibited in Figure 7. The peaks at 3446 and 1630 cm^−1^ belonged to the –OH and the bending mode of absorbed water in the nanocomposites. The adsorptions at 2924 and 890 cm^−1^ are the characteristics of the stretching vibration of C–H groups in pyranoid ring and the β-glycosidic linkages in cellulose. All of the four samples showed the typical bands at 1038 cm^−1^, which is attributed to the C–O–C in cellulose or *v*_3_ PO_4_^3−^ in HA, and the bending mode of *v*_4_ PO_4_^3−^ at 604 and 563 cm^−1^. The peaks at 1463 cm^−1^ (*v*_3-3_ CO_3_^2−^), 1422 cm^−1^ (*v*_3-4_ CO_3_^2−^) and 871 cm^−1^ (*v*_2_ CO_3_^2−^) suggest the existence of calcite, or the [PO_4_^3−^] being partly replaced by [CO_3_^2−^] from the urea. The weakening of these three peaks (1463, 1422, and 871 cm^−1^) in the cellulose/HA nanocomposites prepared using NaH_2_PO_4_·2H_2_O at 180 °C for 10 min (Figure 7a) indicate that the HA nanostructures were purer than the product prepared at 150 °C for 10 min (Figure 3a). Combined with the results from XRD (Figure 6), one can conclude that the higher reaction temperature favored the formation of HA in the composites using inorganic (NaH_2_PO_4_·2H_2_O) or organic (ATP and CP) phosphorus sources, except for FBP.

**Figure 8:**
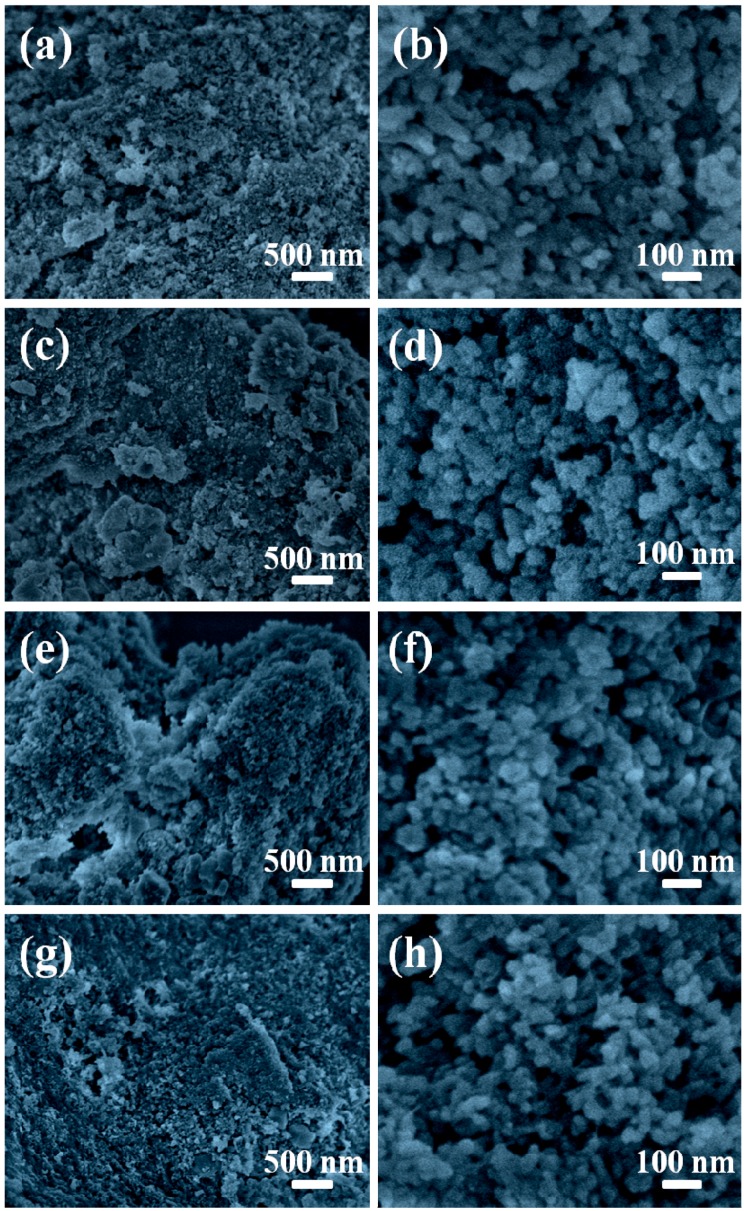
FE-SEM images of the cellulose/HA nanocomposites prepared by microwave-assisted hydrothermal method at 180 °C for 10 min using different phosphorus sources: (**a**, **b**) NaH_2_PO_4_·2H_2_O; (**c**, **d**) ATP; (**e**, **f**) CP; (**g**, **h**) FBP. The magnifications for the low-magnification images on the left column are 20,000 and 100,000 times for the high-magnification images on the right column.

Figure 8 exhibited the morphologies of the cellulose/HA nanocomposites prepared at high temperature (180 °C). The HA nanorods with 57.6 ± 4.2 nm in length and 25.2 ± 3.2 nm in diameter were obtained using NaH_2_PO_4_·2H_2_O as phosphorus source at 180 °C (Figure 8a,b). When using ATP as the organic phosphorus source (Figure 8c,d), the HA appeared as pseudo-spherical structure (with diameter of 22.8 ± 3.8 nm) in the composites. The nanospheres with diameter of 24.6 ± 3.8 nm were obtained using CP as phosphorus source (Figure 8d,f). When using FBP (Figure 8g,h), the HA nanostructures consisted of short nanorods (*l*: 31.7 ± 6.8 nm, *d*: 23.2 ± 4.0 nm) and nanospheres (22.1 ± 1.8) with smooth surfaces. On the contrast, the nanorods with coarse surfaces were obtained when the sample was prepared at 150 °C (Figure 4i,j). These results indicate that the reaction temperature affected the size and morphology of HA in the cellulose/HA nanocomposites, and the HA nanostructure with a smaller size and more regular structure could be obtained by increasing the reaction temperature.

## 4. Conclusions

**Figure 9:**
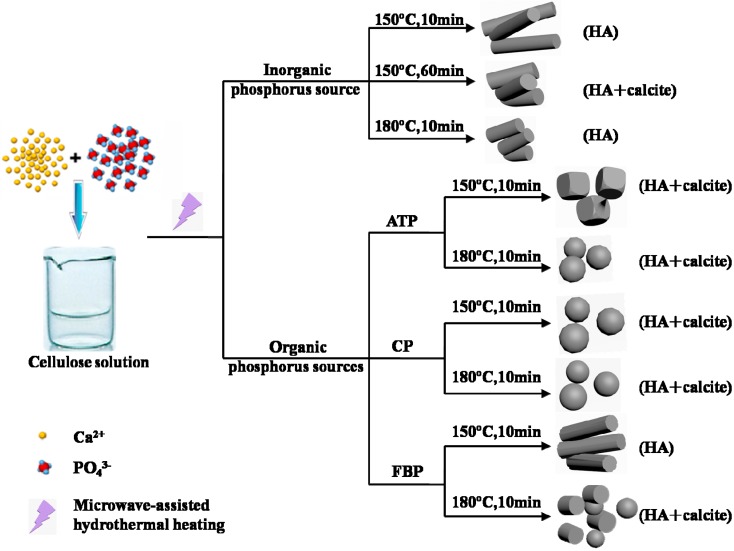
Schematic illustration of the synthetic strategies and results for the cellulose/HA nanocomposites using inorganic (NaH_2_PO_4_·2H_2_O) and organic (ATP, CP, and FBP) phosphorus sources through microwave-assisted hydrothermal method.

In summary, the cellulose/HA nanocomposites were, for the first time, synthesized by using NaH_2_PO_4_ as inorganic phosphorus source, and biomolecules (ATP, CP, and FBP) as organic phosphorus sources through the microwave-assisted hydrothermal method. The experimental results showed (Figure 9): (1) The phosphorus sources played a vital role on the crystal phase, size, and morphology of the minerals in the cellulose/HA nanocomposites. The pure HA could be obtained by using NaH_2_PO_4_·2H_2_O as phosphorus source, while all the ATP, CP, and FBP could lead to the byproduct, calcite. (2) The higher reaction temperature favored the formation of HA with smaller size and more regular structure using inorganic (NaH_2_PO_4_·2H_2_O) or organic (ATP and CP) phosphorus sources. The as-prepared cellulose/HA nanocomposites with different HA morphologies may have potential applications in many fields, such as water treatment, bone tissue engineering, and protein adsorption. 

## Figures and Tables

**Table 1:** The experimental parameters for the preparation of typical cellulose/HA nanocomposites, and their morphologies, Ca/P molar ratios, and the components of the mineral in the nanocomposites.

Sample No.	Phosphorus source	Temp. (°C)/Time (min)	The phase of mineral	Ca/P molar ratio	Morphology
1	NaH_2_PO_4_·2H_2_O	150/10	HA	1.82	Nanorods (*l*: 97.1 ± 9.4 nm, *d*: 21.6 ± 2.8 nm)
2	NaH_2_PO_4_·2H_2_O	150/60	HA, Calcite	2.00	Short nanorods (*l*: 61.2 ± 4.2 nm, *d*: 33.1 ± 4.1 nm)
3	ATP	150/10	HA, Calcite	2.63	Pseudo-cubic (Size: 48.1 ± 4.3 nm)
4	CP	150/10	HA, Calcite	2.32	Pseudo-spherical (*d*: 28.8 ± 3.1 nm)
5	FBP	150/10	HA	1.87	Nanorods (*l*: 69.4 ± 9.3 nm, *d*: 26.9 ± 1.7 nm)
6	NaH_2_PO_4_·2H_2_O	180/10	HA	1.81	Short nanorods (*l*: 57.6 ± 4.2 nm, *d*: 25.2 ± 3.2 nm)
7	ATP	180/10	HA, Calcite	2.33	Pseudo-spherical (*d*: 22.8 ± 3.8 nm)
8	CP	180/10	HA, Calcite	2.17	Nanospheres (*d*: 24.6 ± 3.8 nm)
9	FBP	180/10	HA, Calcite	2.07	Nanorods (*l*: 31.7 ± 6.8 nm, *d*: 23.2 ± 4.0 nm) and Nanospheres (*d*: 22.1 ± 1.8 nm)

HA: hydroxyapatite, Ca_10_(PO_4_)_6_(OH)_2_; Calcite: CaCO_3_; *l*: the length of the nanorods; *d*: the diameter of the pseudo-spherical and nanospheres, or the widest part of the nanorods. The sizes of HA nanoparticles in the nanocomposites were obtained from field emission scanning electron microscopy (FE-SEM) images using Image J software.
